# Comparing the Effects of Oral HIV Self-Testing With Those of Standard HIV Testing for Men Who Have Sex With Men (MSM): A Systematic Review and Meta-Analysis

**DOI:** 10.7759/cureus.28157

**Published:** 2022-08-19

**Authors:** Shruti Vashisht, Shreya Jha, Nishakar Thakur, Anwita Khaitan, Sanjay Rai, Partha Haldar, Shashi Kant, Priyanka Kardam, Meenu Sangral

**Affiliations:** 1 Centre for Community Medicine, All India Institute of Medical Sciences, New Delhi, New Delhi, IND; 2 Department of Community Medicine, Jawaharlal Institute of Postgraduate Medical Education and Research, Pondicherry, IND

**Keywords:** meta-analysis, oral hivst, systematic review, hiv self-test, hiv test

## Abstract

The WHO recommends HIV self-testing (HIVST) as an innovative strategy and an additional testing approach to attain UNAIDS targets to end HIV by 2030. HIVST is a process whereby a person collects his or her own specimen (either oral fluid or blood), performs an HIV test, and interprets the result. It has been described as a discreet and convenient way to reach the hidden, unreached key populations (KPs) who do not know their HIV status or do not get tested. Among the KPs, men who have sex with men (MSM) is one such group that by far remains hidden due to feared stigma and discrimination associated both with their sexuality and HIV. Fear of pain and blood while HIV testing also deters MSM from getting tested. In this review, we assessed the effect of oral HIVST on the uptake and frequency of testing and risk behavior as compared to standard HIV testing.

For this review, we systematically searched various electronic databases for clinical trials comparing HIVST to standard HIV testing from January 1, 2011, to December 31, 2021. A meta-analysis of studies was conducted using a random-effects model for relative risks (RRs) and 95% confidence intervals (CIs). The protocol was registered with PROSPERO, and PRISMA guidelines for systematic reviews and meta-analyses were followed. The quality of the clinical trials was assessed using Cochrane’s risk of bias tool version 2.0 (RoB 2.0).

We identified eight studies comparing HIVST to standard HIV testing services (HTSs). The eight randomized controlled trials (RCTs) enrolled 5,297 participants, of which 5,212 were MSM and 85 were transgender (TG) women. Seven RCTs were conducted in high-income countries (HICs): four in the USA, two in Australia, and one in Hong Kong. One was conducted in a low-middle-income country (LMIC) in Myanmar.In all the studies, HIVST intervention was provided with oral HIVST kits, except for one study in which both blood-based and oral HIVST kits were used. Meta-analysis (five RCTs) showed that HIVST increased the uptake of HIV testing by 1.43 times compared to standard of care (SoC) (RR = 1.43; 95% CI = 1.25, 1.64). Meta-analysis (four RCTs) found that HIVST increased the mean number of HIV tests by 2.34 during follow-up (mean difference = 2.34; 95% CI = 1.66, 3.02). Meta-analysis (four RCTs) showed that HIVST doubled the detection of new HIV infections among those tested (RR = 2.10; 95% CI = 1.35, 3.28) and reported higher repeat testing as compared to the control arm (RR = 2.04; 95% CI = 1.22, 3.42). A meta-analysis of three trials found no significant difference in risk behavior in respect of condomless anal intercourse (CAI) (odds ratio (OR) = 0.90; 95% CI = 0.67, 1.22) and multiple male partnership (RR = 0.89; 95% CI = 0.83, 0.94).

Oral HIVST could increase the HIV testing and detection of new HIV infections among MSM who may not otherwise test, as compared to standard testing services alone. However, further research from low-middle-income countries is required for generalizability.

## Introduction and background

Regular testing is recognized as a key strategy for HIV control. Awareness of one’s HIV-positive status results in the reduction of risky sexual practices, early linkage to care resulting in the early initiation of antiretroviral therapy, and a substantial reduction in the risk of HIV transmission to sexual partners [1]. HIV testing services (HTSs) have scaled up significantly in the past decade [2]. Despite the remarkable progress in the global HIV response, new HIV infections and AIDS-related deaths remain unacceptably high. Only eight countries have achieved the 90-90-90 testing and treatment targets. Globally, in 2020, 1.5 million were newly infected with HIV, 84% (31.6 million) of people living with HIV knew their HIV status, 73% (27.4 million) were accessing treatment, and 66% (24.8 million) were virally suppressed [3].

Focus now shifts to those who have been missed for various reasons such as existing inequalities, barriers to HIV care and testing services, unjust legal laws, stigma, and more shift to virtual platforms during the COVID-19 pandemic. Noticeable among these are men who have sex with men (MSM) who are largely hidden because of the pervasive stigmatization, discrimination, and criminalization of homosexuality. The fear of being outcasted and ridiculed by family members, relatives, and society prevents them from opening up about their sexual identity and links to the prevention, care, support, and treatment services [1]. Enough evidence is available that suggests that psychosocial barriers such as social stigma and discrimination, logistic barriers such as long waiting times, and personal barriers such as the low self-perceived risk of HIV acquisition and inconvenience due to fear of pain in blood tests prevent many MSM from getting tested for HIV at standard of care (SoC) health settings [4].

New technologies and service models in HIV testing are required to reach the unreached and ensure optimal testing rates. HIV self-testing (HIVST) has the potential to increase accessibility to and uptake of HIV testing, particularly among populations that are unreachable by conventional health services. HIVST is a process whereby a person can collect his/her own sample (oral fluid or blood), conduct the test, and interpret the results, alone or in the company of a trustworthy individual. There are four WHO prequalified HIVST products, of which three (Mylan HIVST, INSTI HIVST, and Sure HIVST) are blood-based and have a sensitivity ranging from 97% to 99.8% and specificity ranging from 99.5% to 100%. The OraQuick HIV self-test is an oral fluid-based HIV testing product that has a sensitivity of 100% and specificity of 99.2%. The WHO in 2016 recommended HIVST as a strategy to expand HIV testing services (HTSs), particularly to high-risk and underserved populations, and came up with an updated policy brief in 2019 where it recommends the distribution of HIVST kits by HIV-positive and HIV-negative clients to their partners and contacts [5]. Policy regarding HIVST and the legal framework to include it in national HTS efforts vary according to country. High-income countries, such as the USA and UK, introduced over-the-counter (OTC) sales in early 2012 and 2014, respectively. France and Ireland subsequently implemented their HIVST policies. Among African countries, Kenya was the first to develop its HTS national policy to include the oral HIVST kit in 2008. In SA, the guidelines were developed in 2018 [6]. As of June 2019, 38 countries only had implemented HIVST of the 77 countries, which had supportive policies for HIVST [2].

HIVST innovations offer an important opportunity to reduce stigma and confidentiality concerns among hard-to-reach populations [7].﻿ The evidence across the globe suggests that HIVST offers confidentiality to the users and is thus a fear-free and stigma-free HIV testing. Reviews conducted reported that HIVST may increase the uptake of HIV testing MSM [2,8,9]. The reviews included study designs from observational to RCTs.

In India, HIVST is not yet available, and no policy or guideline exists for the use of HIVST. A recent study that conducted mapping and size estimation of MSM in virtual platforms in New Delhi, India, cited ﻿that 47% of MSM in India have never been tested for HIV [10]. Individuals who are unaware of their HIV status have a transmission rate of 3.5 times higher than individuals who are aware of their status. To improve HIV testing for MSM, HIVST can play a significant role.

This review and meta-analysis aims to understand the effect of oral HIVST on the frequency of HIV testing and the risk behaviors of MSM. The objective of this review is to compare the effects of oral HIVST with those of standard HIV testing. We hope to provide substantial evidence that may help in developing policy guidelines for the introduction of oral HIVST in India.

## Review

We conducted this systematic review in line with the PRISMA guidelines for systematic reviews and meta-analyses [11]. The protocol was registered with PROSPERO on August 9, 2021 (registration number CRD42021261875).

Eligibility criteria

For our review, we followed the PICO question (Table 1). Only cluster randomized/randomized controlled trials (RCT) that compared oral or oral- and blood-based HIVST with standard or any other model of HIV testing among MSM in any global setting were included. Only studies that focused on the desired outcomes and provided quantitative results were selected for the review. Full text and abstracts or posters elaborating any one of the desired outcomes were included. No restrictions were placed on the language search. Qualitative studies, modeling studies, trial protocols, reviews, and study designs other than cluster RCT or RCT were excluded. Studies covering key populations (KPs), but not including MSM, were also excluded from the review.

**Table 1:** Review PICO question HIVST: HIV self-testing

PICO question	
Population	Men who have sex with men (MSM)
Intervention	Intervention that provides oral HIV self-testing or oral- and blood-based HIVST
Comparison	Standard HIV testing services or any other form of HIV testing services
Outcome	HIV testing uptake, HIV testing frequency, new HIV infections detected, risk behavior - condomless anal intercourse and linkage to care

Information sources

The following electronic databases were searched for full-text articles and abstracts: MEDLINE, Embase, Cochrane Library, and Directory of Open Access Journals (DOAJ). Ongoing and completed clinical trials were searched for at ClinicalTrials.gov, WHO ICTRP, and the Clinical Trials Registry of India. The search was conducted from January 1, 2011, to December 31, 2021. Other open-access sites such as medRxiv, Open Knowledge Repository, and BRAC were also searched. We also conducted secondary reference searching on the studies included in the review. Relevant conference abstracts published in the proceedings of the International AIDS Conference (IAC), International AIDS Society Conference, and Conference on Retroviruses and Opportunistic Infections (CROI) (2018-2021) were searched.

Search strategy

The following search strategy was adopted for PubMed and other electronic databases: ((HIV 1) OR (HIV)) OR (HIV2) OR (human immune deficiency virus) OR (HIV type 1) OR (HIV type2 (Title/Abstract)) OR (human immunodeficiency virus (Title/Abstract)) OR (human immune-deficiency virus (Title/Abstract)) OR (acquired immunodeficiency syndrome (Title/Abstract)) OR (acquired immune deficiency syndrome (Title/Abstract)) AND (dried blood spot (Title/Abstract)) OR (dried blood spot self (Title/Abstract)) OR (dried blood spot home (Title/Abstract)) OR (dried blood spot personal (Title/Abstract)) OR (dried blood spot remote (Title/Abstract)) OR (DBS (Title/Abstract)) OR (Oral fluid based test (Title/Abstract)) OR (Saliva based test self (Title/Abstract)) OR (saliva based community (Title/Abstract)) OR (saliva based online (Title/Abstract)) AND (standard of care (Title/Abstract)) OR (Western blot (Title/Abstract)) OR (confirmatory test (Title/Abstract)). For the Cochrane Library, the search strategy was HIV home-based testing in the abstract or HIV rapid test in the abstract OR HIV oral fluid-based test in the title, abstract, and keyword and MSM in the title, abstract, and keyword or men who have sex with men in the title, abstract, and keyword (word variations have been searched). Four reviewers (SV, NT, SJ, and AK) evaluated and assessed citations for eligibility and assessed the quality. A senior reviewer (SK) was consulted on citations for the resolution of discordance.

Data abstraction

The first author (SV) and the study team members (NT, SJ, and AK) independently abstracted all data on the following items: study design, study population, sample size, outcome measures (uptake and frequency of HIV testing, new HIV infection detected, and risk behavior - condomless sexual intercourse (CAI) and proportion linked to care). A senior reviewer (SK) was consulted for the resolution of discrepancies in data abstraction.

The primary outcomes used to compare the oral HIVST with standard HTS were as follows: uptake of HIV testing services (defined as the proportion of people offered HIV testing who accepted and completed any HIV testing in a specified time frame) and frequency of HIV testing (defined as the mean number of HIV tests conducted during a specified time frame). We also assessed secondary outcomes that included the proportion of newly diagnosed HIV positive (defined as HIV infections detected during the specified time frame), sexual risk behavior (CAI (defined as condomless sex with partners) and multiple male sex partners), and repeat HIV testing (defined as more than two tests conducted in the follow-up period).

Data analysis

﻿A meta-analysis was conducted where the same or a comparable outcome was reported by two or more studies. It was conducted using random-effects models in RevMan version 5.4. For binary outcomes, the number of events was calculated and pooled. For continuous outcomes, the mean and SD were calculated and pooled. Statistical heterogeneity was evaluated. When outcomes were measured and reported at multiple time points, we used the longest time point or the end of the study period where possible. For two studies that had multiple intervention arms, data from HIVST arms were clubbed together and compared to the control arm as reviewers assessed that the interventions were unlikely to influence the outcome.

Quality assessment

**Figure 1:**
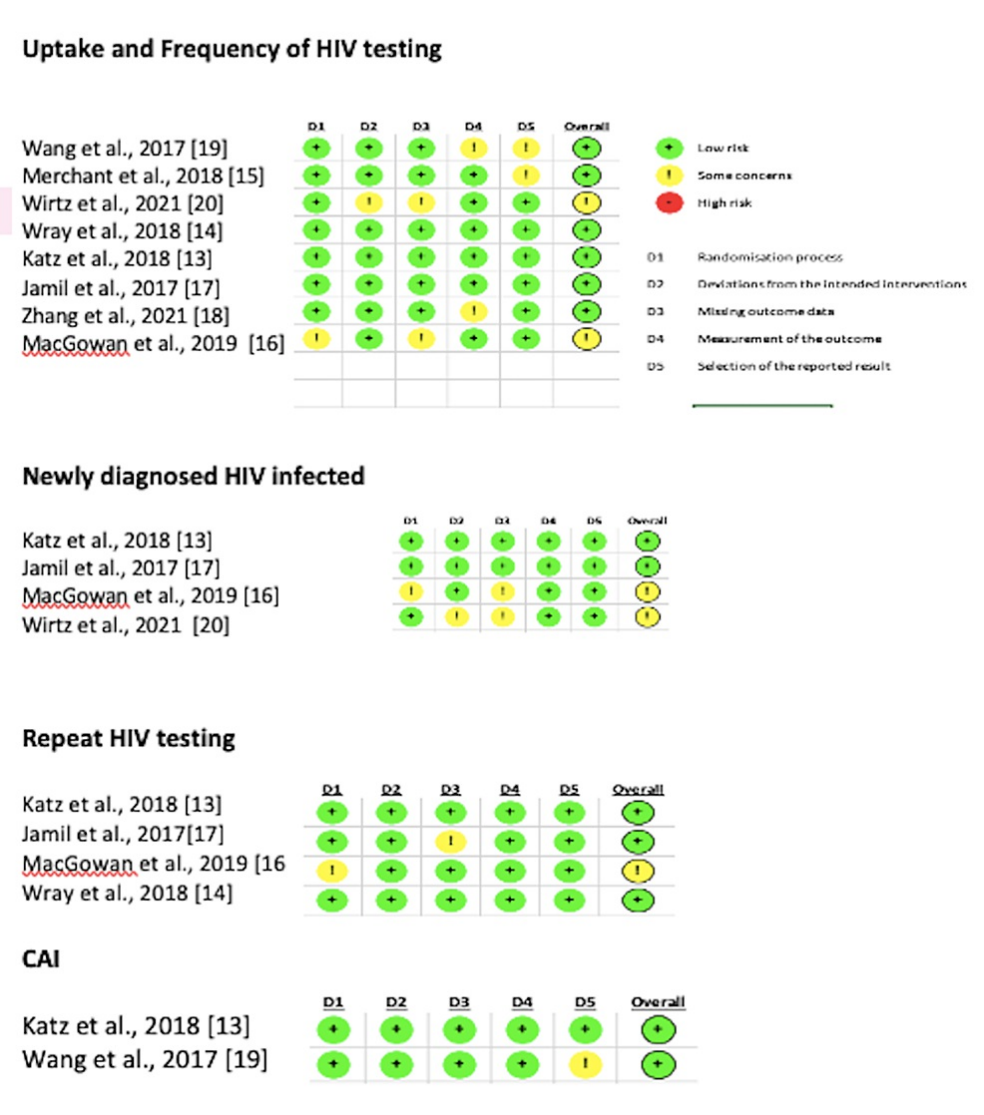
Risk of bias

The quality of the clinical trials was assessed using Cochrane’s risk of bias tool version 2.0 (RoB 2.0) [12]. This included evaluation of risk pertaining to six domains: bias arising from the randomization process, due to deviations from intended interventions, due to missing outcome data, in the measurement of the outcome, in the selection of the reported result, and overall bias (Figure 1).

Results

**Figure 2:**
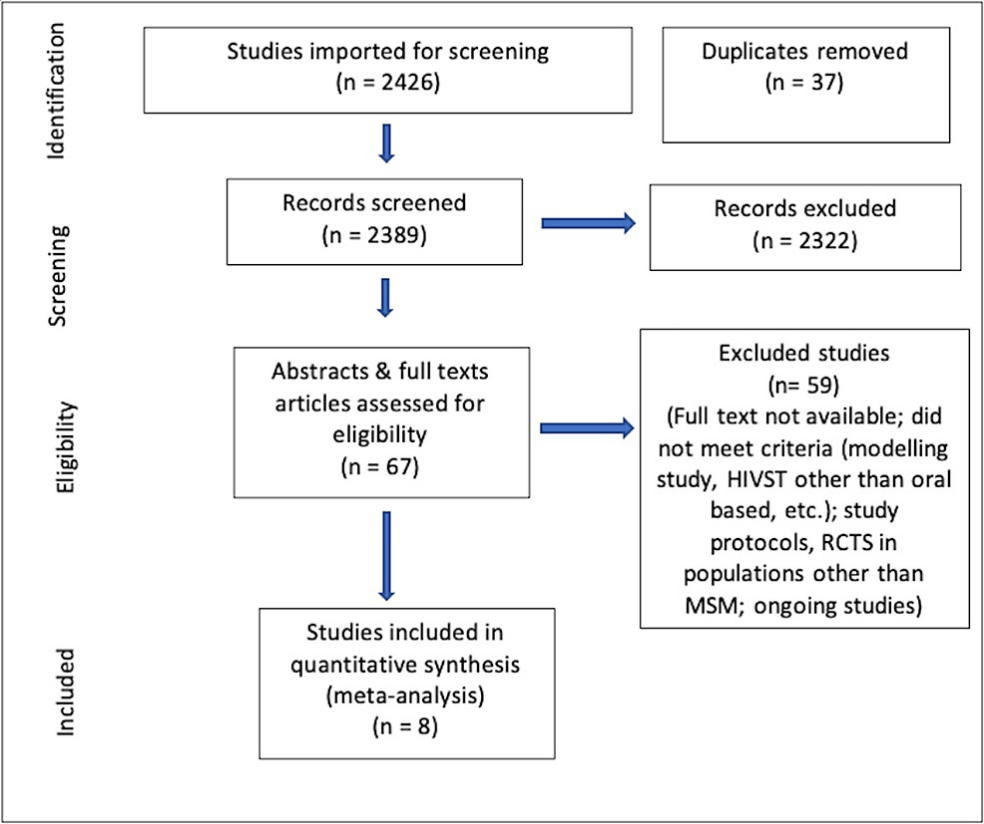
Study selection HIVST: HIV self-testing; RCTs: randomized controlled trials; MSM: men who have sex with men

We could identify 2,426 records from various electronic databases and from other sources. After the removal of duplicates (n = 37), 2,389 records were screened. We excluded 2,322 records, and 67 abstracts and full texts were screened for eligibility, of which 59 were excluded. Eight studies met the inclusion criteria (Figure 2).

The eight RCTs enrolled 5,297 participants, of which 5,212 were MSM and 85 were transgender (TG) women. HIV-negative MSM or those of unknown status only were included in the study. Of the eight studies, seven were conducted in high-income countries (HICs): four in the USA [13-16], two in Australia [17,18], and one in Hong Kong [19]. One was conducted in a low-middle-income country (LMIC) in Myanmar [20]. The characteristics of the included RCTs are shown in Table 2.

**Table 2:** Characteristics of the included RCTs HIVST: HIV self-testing; CAI: condomless anal intercourse; HIVST-OIC: HIVST kit and online real-time instructions and pre-test/post-test counseling; YMSM: young men who have sex with men; STI: sexually transmitted infection

Author, year	Setting	Total randomized	Intervention	Standard of care	Mode of distribution	Test kit	Outcomes
Wang et al., 2017 [19]	Hong Kong	430	Free HIVST-OIC	Traditional HIV testing	Mailed HIVST kit	Oral	Primary outcome: whether the participant had taken up any HIV testing within the six-month follow-up period; secondary outcome: to measure CAI with men and multiple male sex partners in the last three months (among testers only)
Jamil et al., 2017 [17]	Australia	362	HIVST kits and facility-based testing	Facility-based testing	Four HIVST kits were distributed at the study site; additional kits could be picked up from the study site or were mailed	Oral	Primary outcome: to measure the number of HIV tests over 12 months; secondary outcomes: the number of episodes of STI testing (chlamydia, gonorrhea, or syphilis) over 12 months; sexual risk behavior during follow-up; reason and the acceptability of HIV self-testing
Katz et al., 2018 [13]	USA	197	Oral HIVST kits	Standard HIV testing as per national guidelines	Distributed at the study site	Oral	Primary outcome: the number of times tested for HIV; secondary objective: to measure the incidence of CAI, the number of male CAI partners, and STI prevalence
Merchant et al., 2018 [15]	USA	425	First arm: oral fluid rapid HIV self-test; second arm: mail-in blood sample collection	Community organization/medical facility testing	Purchased online by participants	Oral	Primary outcome: comparing the completion of HIV testing within a three-month period in the intervention and control groups; secondary outcome: to measure the use of any, another, or no HIV test; time to HIV test completion; and willingness to refer and referrals of other black, Hispanic, or white YMSM
Wray et al., 2018 [14]	USA	65	First arm: HST with follow-up (eTEST); second arm: HST with no follow-up (standard HST)	Reminders for clinic-based testing	Mailed	Oral	HIV testing, repeat testing, STI testing, counseling for risk reduction, receiving condoms and lube, risk behavior - condomless anal sex
MacGowan et al., 2019 [16]	USA	2,665	HIVST kits	Standard HIV testing as per national guidelines	Two oral and two blood-based kits were mailed	Oral and blood	Primary outcome: to measure the frequency of HIV testing and the number of newly identified HIV infections; secondary outcome: any HIV testing, HIV testing reported on at least three follow-up surveys, provider-based testing, testing among those who had never been tested at enrollment, linkage to care, sexual behavior (male anal sex partners, male anal sex partners without using condoms, total number of sex partners, and serosorting)
Zhang et al., 2021 [18]	Australia	279	HIVST kits and facility-based testing	Facility-based testing in the first year, after which all had access to HIVST kits	Distributed at the study site	Oral	Primary outcome: overall frequency of HIV tests (both HIVSTs and facility-based tests) in any 12-month period in years 1 and 2
Wirtz et al., 2021 [20]	Myanmar	577	Unassisted HIVST	Community-based HIV testing	Distributed at the study site	Oral	HIV testing, undiagnosed infections, challenges with HIV testing

In all the studies, HIVST intervention was provided with oral HIVST kits, except in one study in which both blood-based and oral HIVST kits were used [16]. All studies compared HIVST with standard HTS. The HIVST kits were either distributed at the study site (n = 4) [13,17,18,20] or mailed (n = 3) [14,16,19] to the participants. In one study, the participants were provided with a weblink to purchase the kits online [15]. The minimum age of participants was 18 years. Pre-test and post-test counseling were provided to the study participants through various modes (Table 3).

**Table 3:** Counseling of the study participants NI: no information

Author, year	Assisted/unassisted HIVST	Pre-test Counseling	Post-test counseling	Linkage to care
Wang et al., 2017 [19]	Assisted	Provided by the administrators (10-15 minutes)	Provided by the administrators (15-25 minutes)	The participants who received a positive HIV test were accompanied by the research staff to the NGOs and/or Department of Health
Jamil et al., 2017 [17]	Unassisted	24-hour telephone support line	24-hour telephone support line	The participants who received a reactive self-test were offered expedited confirmatory testing, clinical review, and supportive counseling at the study clinics
Katz et al., 2018 [13]	Unassisted	Printed material and 24-hour telephone support line	Printed material and 24-hour telephone support line	24-hour telephone support line
Merchant et al., 2018 [15]	Unassisted	NI	NI	NI
Wray et al., 2018 [14]	Unassisted	Risk reduction counseling offered during follow-up calls	NI	NI
MacGowan et al., 2019 [16]	Unassisted	Online link to AIDSvu.org Study telephone number (9 am to 5 pm EST), Monday to Friday; mental health counseling after hours and on weekends	Online link to AIDSvu.org Study telephone number (9 am to 5 pm EST), Monday to Friday; mental health counseling after hours and on weekends	Online link to AIDSvu.org Study telephone number (9 am to 5 pm EST), Monday to Friday; mental health counseling after hours and on weekends
Zhang et al., 2021 [18]	Unassisted	24-hour telephone support line	24-hour telephone support line	The participants who received a reactive self-test were offered expedited confirmatory testing, clinical review, and supportive counseling at the study clinics
Wirtz et al., 2021 [20]	Unassisted Telephone hotline for any assistance, if required	Provided by the administrators	Provided by the administrators, telephone hotline	﻿Linkages to HIV care and future HIV testing for those with negative results were made to community-based affirming health facilities

Uptake of HIV Testing

**Figure 3:**
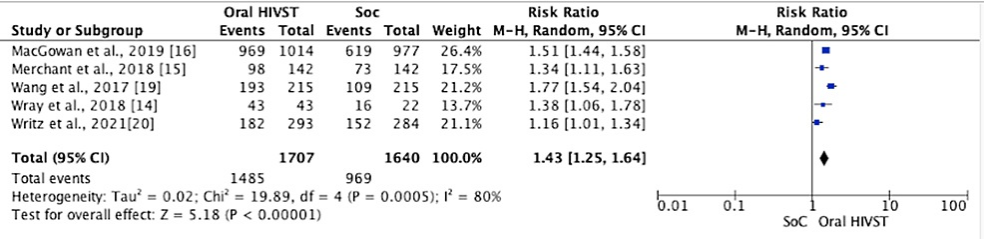
Forest plot comparing the uptake of HIV testing in oral HIVST as compared to standard of care HIVST: HIV self-testing; SoC: standard of care; CI: confidence interval

﻿Five of the eight RCTs reported the uptake of HIV testing. A meta-analysis showed that HIVST increased the uptake of HIV testing by 1.43 times compared to SoC (relative risk (RR) = 1.43; 95% confidence interval (CI) = 1.25, 1.64; Tau2 = 0.02; Chi2 = 19.89; df = 4; I2 = 80%). All the five RCTs showed an increase in the uptake of HIV testing, which was statistically significant (p-value = 0.0005) (Figure 3). Wirtz et al. [20] reported a 76% increase in testing rates across both arms, ﻿relative to baseline lifetime HIV testing history. In the study by Wang et al. [19], high uptake of any type of HIV testing was reported in the HIVST group at month 6 (89.8% versus 50.7%; RR = 1.77; 95% CI = 1.54, 2.03; NNT = 2.56; 95% CI = 2.13, 3.20; p = 0.001). The results remained statistically significant in subgroup analysis based on with and without CAI, multiple male sex partners, and experience in HIV testing in the last three years. Wray et al. [14] reported 100% testing for HIV at some point during the study period using any test in the intervention arms as compared with 72% of control participants, a statistically significant between-group effect (F(2, 62) = 7.69; MS = 0.54; p = 0.001). The study by Merchant et al. [15] reported that 59% of the participants in the HIVST arm completed any type of HIV test (54% completed their assigned test, and 5% used a test they were not assigned), and 41% were not tested. However, in their study, the completion of the assigned HIV test was greater in the oral fluid rapid HIV self-test and the community organization/medical facility arms than in the mail-in blood sample collection HIV test arm (p < 0.01 for both comparisons). MacGowan et al. [16] reported a 55.7% increase in annual HIV testing among HIVST participants as compared to only a 6.9% increase in the control arm.

Frequency of HIV Testing

**Figure 4:**
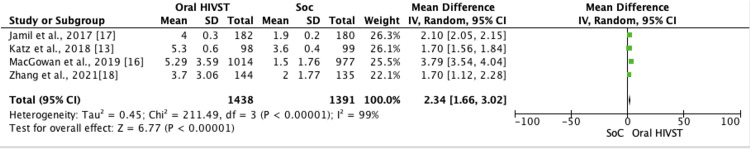
Forest plot comparing the frequency of HIV testing in oral HIVST as compared to standard of care HIVST: HIV self-testing; SoC: standard of care; CI: confidence interval

A meta-analysis of four RCTs found that the mean number of HIV tests increased by 2.34 during follow-up in the HIVST arm (mean difference = 2.34; 95% CI = 1.66, 3.02) (Figure 4). Three studies delivered HIVST through facility distribution (with additional optional mail distribution) and had smaller effect sizes at 2 [14] and 1.7 [15,18], respectively. According to Jamil et al. [17], HIV testing in the HIVST group was significantly greater than in the SoC group (RR = 2.08; 95% CI = 1.82, 2.38; p < 0.0001). Zhang et al. [18] reported that the mean overall HIV testing frequency per person in year 2 among men in the intervention arm was higher than in year 1 of the SoC arm (3.7 versus 2.0; RR = 1.71; 95% CI = 1.48, 1.97; p < 0.001). In the study by Katz et al. [13], MSM randomized to the HIVST arm reported significantly more HIV tests during follow-up (mean = 5.3; 95% CI = 4.7, 6.0) than those in the control arm (mean = 3.6; 95% CI = 3.2, 4.0; p < 0.0001). This represented an average increase of 1.7 tests per MSM over 15 months (95% CI = 0.9, 2.5). The study that demonstrated the largest difference at 3.80 was conducted by MacGowan et al. [16]. The HIVST participants reported frequent testing as compared to the control arm (mean number of tests over 12 months: 5.3 versus 1.5; p < 0.001).

New HIV Infections

**Figure 5:**
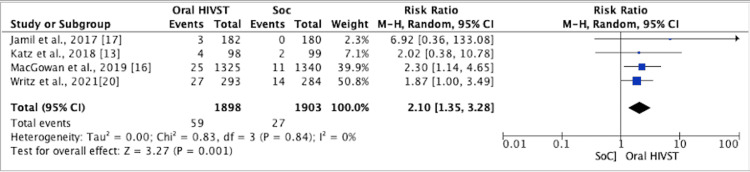
Forest plot comparing new HIV infections in oral HIVST as compared to standard of care HIVST: HIV self-testing; SoC: standard of care; CI: confidence interval

A meta-analysis of four RCTs indicated that oral HIVST had positive effect on the detection of new HIV infections among those tested, and this was statistically significant (RR = 2.10; 95% CI = 1.35, 3.28; I2 = 0%, p = 0.001) (Figure 5). In the study by Katz et al. [13], four in the HIVST arm and two in the SoC arm were HIV-positive. MacGowan et al. [16] reported more than twice as many HIV infections identified in the HIVST arm as compared to the SoC arm (1.9% versus 0.8%; p = 0.02). In the follow-up study by Jamil et al., three men were newly diagnosed with HIV during follow-up [17], and the overall incidence was 0.9 per 100 person-years (95% CI = 0.2, 2.6). All new infections were in the HIVST group. ﻿HIVST identified 27 (15%) previously undiagnosed HIV infections compared to 14 (9%) identified by SoC [17].

Repeat HIV Testing

**Figure 6:**
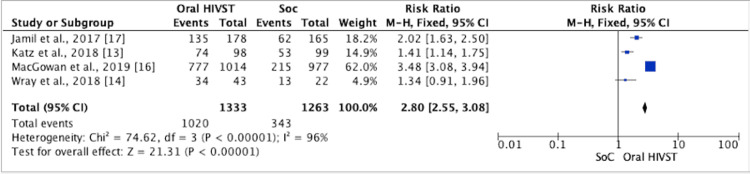
Forest plot comparing repeat HIV testing in oral HIVST as compared to standard of care HIVST: HIV self-testing; SoC: standard of care; CI: confidence interval

We conducted a meta-analysis on four of eight RCTs reporting results for repeat testing. The results of the meta-analysis show that the HIVST arm reported 2.80 higher repeat testing as compared to the control arm (RR = 2.04; 95% CI = 1.22, 3.42; I2 = 96%) (Figure 6). The analysis showed a statistically significant result. Jamil et al. [17] reported more men with more than two HIV tests (76% versus 38%) during follow-up in the HIVST group as compared with the SoC group (p < 0.0001). In the study by Katz et al. [13], MSM in the intervention arm reported three monthly testing as recommended (76% versus 54%, respectively; p = 0.001). Wray et al. suggested significantly different rates of repeat testing across study conditions (F(2, 62) = 5.33; MS = 1.06; p < 0.007) due to lower rates of repeat testing in the control condition (F(2, 62) = 24.5; p < 0.001) [14].

Risk Behavior: Condomless Anal Intercourse

**Figure 7:**
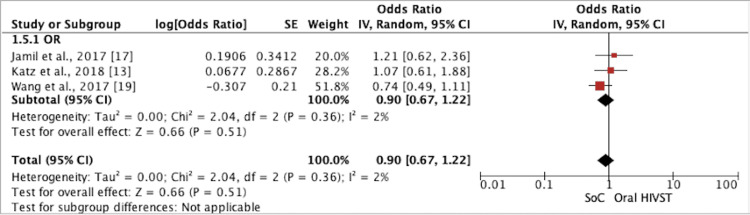
Forest plot comparing condomless anal intercourse among MSM in oral HIVST as compared to standard of care HIVST: HIV self-testing; SoC: standard of care; CI: confidence interval

﻿A meta-analysis of three RCTs found that among MSM, oral HIVST had no statistically significant effect on CAI (odds ratio (OR) = 0.90; 95% CI = 0.67, 1.22; I2 = 2%; p-value = 0.51) (Figure 7). Katz et al. [13] reported that at the nine-month and end-of-study surveys, MSM randomized to HIVST were 1.07 times more likely to report non-concordant CAI in the prior three months than MSM in the control group (OR = 1.07; 95% CI = 0.61, 1.90). Overall, there was no effect on the reduction of sexual risk behavior. Wang et al. [19] also reported no significant difference in CAI (intervention: 27.5% versus control: 33.9%; p = 0.237). Statistically significant reductions in the prevalence of CAI (month 6 versus baseline: p = 0.002) were found within the intervention group only. Jamil et al. [17] reported no statistical association of reporting CAI with casual partners between the HIVST group and the SoC group (OR = 1.21; 95% CI: 0.62, 2.35; p = 0.575). Wray et al. [14] reported no statistical association between the HIVST group and the SoC group after taking into account repeated measures, reporting condomless anal intercourse with casual partners (OR = 1.21; 95% CI = 0.62, 2.35; p = 0.575). This was not included in the meta-analysis as they reported that the results were not comparable.

Risk Behavior: Multiple Male Sex Partners

**Figure 8:**
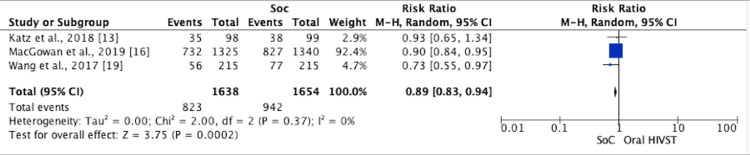
Forest plot comparing multiple male sex partners of MSM in oral HIVST as compared to standard of care HIVST: HIV self-testing; SoC: standard of care; CI: confidence interval

A meta-analysis was conducted on the three trials reporting results for multiple male partnerships in the follow-up period. The overall RR was 0.89 (95% CI = 0.83, 0.94; I2 = 0%) (Figure 8). Wang et al. [19] reported a significant between-group difference at month 6 (intervention: 34.2% versus control: 47.7%; RR = 0.72; 95% CI = 0.54, 0.95; p = 0.021). In the study by Katz et al. [13], HIVST was non-inferior to SoC with respect to multiple male sex partners. At the end of the study surveys, MSM in the oral HIVST arm reported 8% fewer male CAI partners than MSM in the control arm in the prior three months (incidence rate ratio = 0.92; 95% CI = 0.64, 1.33). MacGowan et al. [16] also reported similar results, where no statistically significant difference between participants was observed.

The participants in all the trials who received a reactive result on oral HIVST were linked to care. The participants were contacted, and counseling was provided either by the administrators or on phone by research staff. We also looked for any reported social harms in the trials. We did not find any included trials that studied problems faced with oral HIVST manipulation or unintended harm.

Discussion

HIVST was introduced way back in 2000 by the Joint United Nations Program on HIV/AIDS [9] and had only been implemented in 38 countries by June 2019 [2]. HIVST could play a major role in increasing HIV testing. The studies analyzed in this review were published in the last five years and were limited to MSM. This systematic review and meta-analysis of eight RCTs conducted among MSM found that when compared to SoC, oral HIVST increases the uptake and frequency of HIV testing. The free distribution of HIVST kits can potentially increase the uptake and frequency of HIV testing among MSM. One study, where the participants had purchased the kits, also reported an increase in the uptake of HIV testing [12]. This can result in a reduction in the proportion of MSM unaware of their status due to non-testing for various reasons. The overall outcome may be decreased in new infections as desired for achieving “End AIDS by 2030.” In addition to the increase in the uptake of HIV testing, the meta-analysis suggests that repeat testing for HIV also increased oral HIVST use. This is an important feature of the HIVST strategy, as the prevalence of HIV is 6-13 times higher among KPs, including MSM, in India as compared to the adult prevalence due to their high-risk behavior [21]. Repeat testing 3-4 times a year is recommended by the WHO for high-risk groups (HRGs). Oral HIVST has the potential to increase repeat testing among MSM and thus early detection of the HIV infection.

Four RCTs included in this review assessed the impact of oral HIVST in detecting new infections. A statistically significant positive effect was seen in the analysis. More new infections were diagnosed in the oral HIVST groups as compared to the SoC arm. Thus, more MSM were aware of their status by the introduction of oral HIVST. This is one of the main goals of the 95-95-95 targets, and oral HIVST has the potential to achieve this target. Another study by Okoboi et al. distributed oral HIVST kits through peers and reported identifying eight (5.6%) participants with undiagnosed HIV infection during the three months of follow-up compared to only four (2.7%; p = 0.02) in the SoC arm [22]. The study was not included in the review as it did not match our eligibility criteria.

This increase in HIV testing uptake, repeat testing, and identification of new infections have important public health implications. Approaches to increase regular HIV testing supplemented by oral HIVST could identify infections at an early stage. This would also help attain the WHO recommendations of quarterly testing by high-risk populations, especially MSM. Oral HIVST has the potential of not only increasing the detection of undiagnosed HIV infection but also expanding HIV testing among non-frequent/delayed testers [18].

﻿There was no increase in the sexual risk behavior of MSM as measured by the analysis of CAI with regular or casual partners and multiple male partnerships. None of the studies in the review reported significant differences in CAI and multiple male partnerships between the experimental arm and the control arm. This suggests that the fear of the increase in risk behavior due to oral HIVST is unjustified. It points to the fact that continuous behavioral modification through regular health education and counseling is required to reduce risk behavior among MSM.

Strengths and limitations

In our study, we focused on the effectiveness of oral HIVST kits only among MSM. Evidence has shown that ﻿oral HIVST kits are less painful than finger-prick HIVST kits and are more acceptable to KPs for HIV testing [23,24]. We also included the latest evidence in the literature on oral HIVST.

The limitation of our study is that most of the studies included in the analysis were conducted in HICs. Limited studies from LMICs limit the scope of generalizability of the results to resource-limited settings. The analysis or review of social harm could not be carried out as well, as none of the included trials covered the topic of social harm. Synthesis of results from qualitative studies is required on this topic.

## Conclusions

Our systematic review and meta-analysis suggests that oral HIVST could increase the HIV testing and detection of new HIV infections among the high-risk population of MSM to optimal levels. HIVST could be a major strategy contributing to the 95-95-95 target of the WHO and bringing on track the efforts to end AIDS by 2030. HIVST can be made available in countries where it is not yet available. Programs best suiting the cultural and economic milieu can be implemented to reach those who are at risk and are not aware of their status.
